# Research on the Cell Wall Breaking and Subcritical Extraction of Astaxanthin from *Phaffia rhodozyma*

**DOI:** 10.3390/molecules29174201

**Published:** 2024-09-04

**Authors:** Wenxuan Jiang, Xiangrong Deng, Lanxian Qin, Dahai Jiang, Mengqi Lu, Kai Chen, Manqi Yang, Liangliang Zhang, Jianchun Jiang, Liming Lu

**Affiliations:** 1 Academy of Advanced Carbon Conversion Technology, Huaqiao University, Xiamen 361021, Chinajiangjc@icifp.cn (J.J.); 2 College of Chemical Engineering, Huaqiao University, Xiamen 361021, China; 3 Fujian Provincial Key Laboratory of Biomass Low-Carbon Conversion, Huaqiao University, Xiamen 361021, China; 4 Institute of Chemical Industry of Forest Products, Chinese Academy of Forestry, Nanjing 210042, China

**Keywords:** astaxanthin, *P. rhodozyma*, cell wall breaking, subcritical extraction

## Abstract

This study focused on developing an effective cell wall-breaking method for *Phaffia rhodozyma*, followed by utilizing subcritical fluid extraction to isolate, extract, and concentrate astaxanthin from the complex fermentation products of *P. rhodozyma*. A comprehensive comparison of seven distinct methods for disrupting cell walls, including dimethyl sulfoxide treatment, lactic acid treatment, sodium hydroxide treatment, *β*-glucanase enzymatic digestion, *β*-mannanase enzymatic digestion, and a combined enzymatic treatment involving both *β*-mannanase and *β*-glucanase was conducted. The results identified the lactic acid method as the most effective in disrupting the cell walls of *P. rhodozyma*. The software, Design Expert, was used in the process of extracting astaxanthin from cell lysates using a subcritical extraction method. Through fitting analysis and response surface optimization analysis by Design Expert, the optimal extraction conditions were determined as follows: an extraction temperature of 41 °C, extraction frequency of two times, and extraction time of 46 min. These parameters facilitated the efficient extraction, concentration, and enrichment of astaxanthin from *P. rhodozyma*, resulting in an astaxanthin concentration of 540.00 mg/L. This result can establish the foundation for its high-value applications.

## 1. Introduction

**Figure 1:**
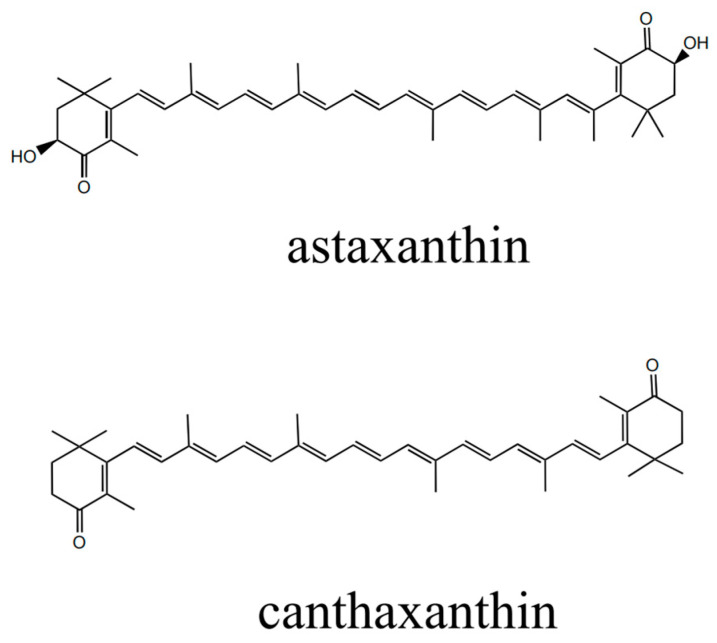
Chemical structures of astaxanthin and canthaxanthin.

Astaxanthin, a ketone carotenoid, has the chemical formula 3,3′-dihydroxy-*β*,*β*′-carotene-4,4′-dione (Figure 1). Astaxanthin is soluble in lipids [1] and organic solvents, but insoluble in water [2]. The structural diagrams of astaxanthin and canthaxanthin can be found in Figure 1. Research has shown that astaxanthin has a range of biological activities, such as anticancer, antioxidant, antiaging, and antihypertensive effects [3]. Additionally, astaxanthin has been recognized for its coloring properties and potential to increase the reproductive rates of animals [4]. The antioxidant capacity of astaxanthin is notably superior to that of lutein by more than 2.8 times. Astaxanthin can also protect cell membranes 100 times more effectively than vitamins can. Furthermore, astaxanthin is 800 times more potent than coenzyme Q and 6000 times more effective than vitamin C in quenching singlet oxygen [4,5,6,7,8]. Consequently, astaxanthin has garnered significant attention from multiple industries and is extensively utilized in feed additives [9], health products [10], cosmetics [11], and pharmaceuticals [12].

The primary sources of astaxanthin include yeast, algae, and shrimp and crab shells as well as chemical synthesis [13]. *Phaffia rhodozyma*, recognized for its safety profile, biological efficacy, and economical production, is the predominant source of natural astaxanthin. However, its astaxanthin content ranges from only 0.02% to 0.04%, which limits its extensive industrial application [14,15]. Therefore, it is imperative to develop a cost-effective and efficient method to separate, extract, and concentrate astaxanthin from the complex metabolic products of *P. rhodozyma*, with the aim of meeting its full potential for higher value applications.

Supercritical CO_2_ fluid extraction (also simply called supercritical fluid extraction) operates on a fundamental principle that hinges on the relationship between the solvency of a supercritical fluid and its density. This relationship is modulated by the application of pressure and temperature, which significantly influence the solvency properties of the supercritical medium. This process involves placing the supercritical fluid into contact with the substance intended for separation while in its supercritical state. This interaction enables selective extraction that discriminates based on the polarity, boiling points, and molecular weights of the components, thereby allowing sequential and controlled extraction of the desired constituents [16].

Subcritical propane has been successfully employed to extract sesame oil from microwaved sesame seeds [17]. Additionally, subcritical water has been utilized effectively to extract phenolic compounds from onion skin waste [18] and to extract rosmarinic acid from lemon honeysuckle [19]. However, reports on the utilization of this method for extracting astaxanthin from *P. rhodozyma* are lacking.

The aim of this study was to identify the most effective methods for lysing the cell wall of *P. rhodozyma* and establish the optimal conditions for astaxanthin removal by subcritical extraction.

## 2. Results

### 2.1. Construction of the Astaxanthin Standard Curve

The standard curve for astaxanthin was determined as described in Section 3.5.3, yielding the equation Y = 0.0081X − 0.2717, with a coefficient of determination (R^2^) of 0.99.

### 2.2. Astaxanthin Content Obtained by Different Wall Disruption Methods

**Figure 2:**
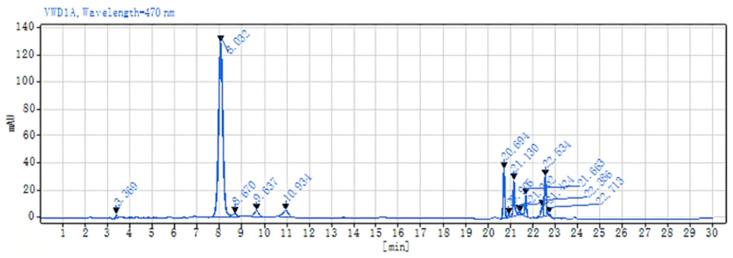
Chromatogram of astaxanthin extracted from *P. rhodozyma* using the lactic acid method.

The astaxanthin content in the samples derived from seven distinct cell wall disruption methods was meticulously analyzed in accordance with the protocol detailed in Section 3.5.4, and the findings are presented in Table 1. The lactic acid method provided the most astaxanthin after the cell wall disruption, followed by the NaOH method at 40 °C for 2 h and the DMSO method. The chromatographic profile of the astaxanthin extracted via the lactic acid method is shown in Figure 2.

### 2.3. Subcritical Extraction

#### 2.3.1. Optimization of the Subcritical Extraction Method

The extracted raw material was the product of *P. rhodozyma* breakdown via the lactic acid method, and the amount of extracted astaxanthin was determined by the method described in Section 3.5.4. The extraction temperature (A), extraction frequency (B), and extraction time (C) were selected for RSM analysis using the astaxanthin content (Y) as the response value. In accordance with the Box–Behnken design, a three-factor and three-level experiment was conducted. The experimental design and results are shown in Table 2.

Using the Design Expert data analysis software, multivariable regression fitting was performed on the three-factor and three-level data in Table 3, and the obtained quadratic regression model equation was Y = −3514.1 + 108.24 × A + 480.825 × B + 52.76167 × C − 2.25 × AB − 0.10667 × AC − 0.38333 × BC − 1.20175 × A^2^ − 90.925 × B^2^ − 0.51744 × C^2^.

The results in Table 4 show that the model F value is 30.26 and *p* < 0.0001, indicating that the established model reached significance (*p* < 0.0001), and the difference in the lack-of-fit was not significant. Additionally, R^2^ = 0.99 suggests that the obtained model fits the data well and could be used for predictive analysis. The F values of each factor in Table 4 show that the degree of influence of each factor on the astaxanthin content was as follows: A (extraction temperature) > C (extraction time) > B (extraction frequency). There were certain interactions between the extraction temperature and extraction frequency (AB), extraction temperature and extraction time (AC), and extraction frequency and extraction time (BC), which significantly affected the content of astaxanthin.

**Figure 3:**
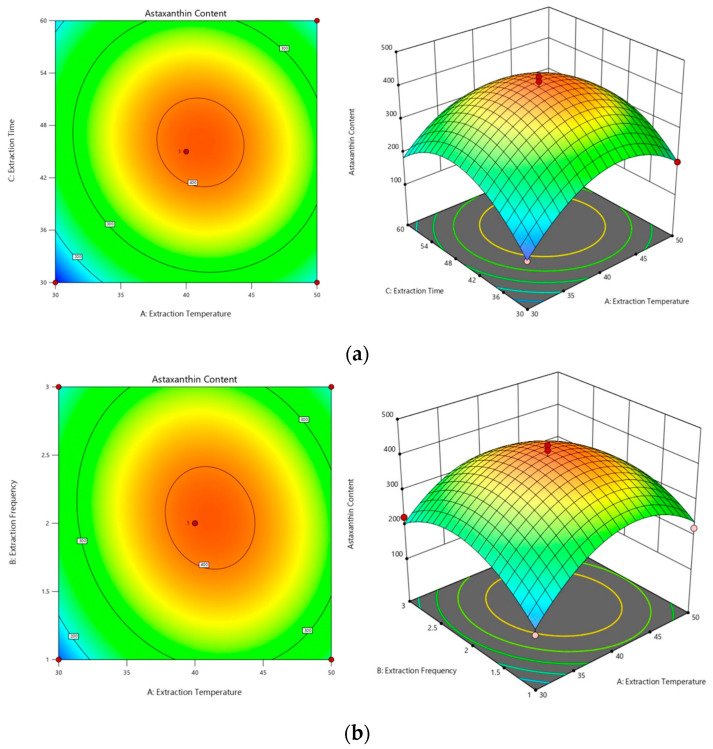

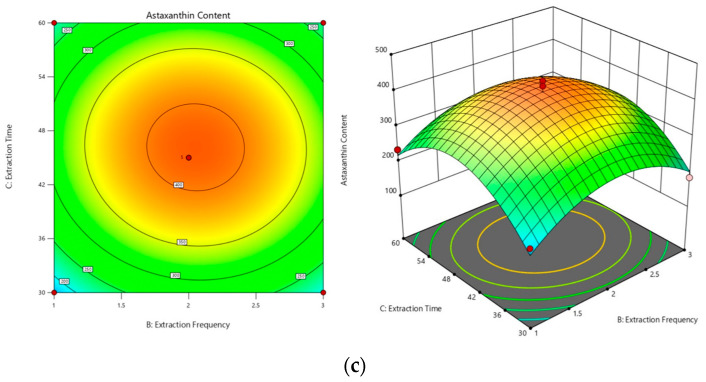
Three-dimensional contour plots and response surface maps. (**a**) Surface and contour plots of the effect of the interaction of extraction temperature and extraction time on astaxanthin content (AC). (**b**) Surface and contour plots of the effect of the interaction of extraction temperature and extraction frequency on astaxanthin content (AB). (**c**) Surface and contour plots of the effect of the interaction of extraction time and extraction frequency on astaxanthin content (BC).

Response surface plots were generated with the regression model to elucidate the interactive effects of pairs of factors on the astaxanthin content. The curvature of the response surface in these plots indicates the degree of influence of each pair, with a more pronounced curvature corresponding to a more significant change in the response value with varying factors. As illustrated in Figure 3, the influence of factor interactions on the proportion of astaxanthin was ranked as follows: extraction temperature and time (AC) (Figure 3a) had a greater impact than did temperature and the number of extractions (AB) (Figure 3b), which in turn had a greater impact than did the interaction between the number of extractions and time (BC) (Figure 3c). The contour plots illustrate the strength of these interactions, with more elliptical shapes suggesting steeper response surfaces and consequently a more pronounced effect on astaxanthin content.

#### 2.3.2. Optimal Process Conditions and Validation Experiments

The model equation was utilized to determine the optimal extraction parameters, which were determined to be an extraction temperature of 41.08 °C, an extraction frequency of 2.04, and an extraction duration of 45.99 min, and should yield an astaxanthin concentration of 412.885 mg/L. To enhance practicality, these optimal conditions were refined to an extraction temperature of 41 °C, with the frequency standardized to two extractions and the time rounded to 46 min. Validation experiments were then conducted in triplicate under these refined conditions, and the mean value was determined to ensure the robustness of the astaxanthin concentration results. The resulting astaxanthin content was 406.54 mg/L, which closely aligns with the value predicted by the model, suggesting high model accuracy and demonstrating the practical feasibility of the optimized conditions.

### 2.4. Astaxanthin Content Detection Results

#### UPLC-Q-TOF MS Detection of Astaxanthin

**Figure 4:**
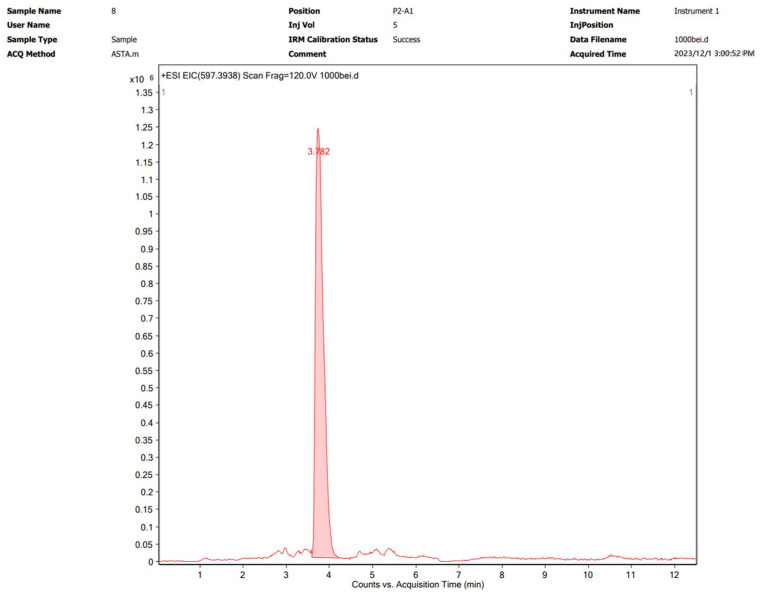
UPLC chromatogram of an astaxanthin sample from *P. rhodozyma* after subcritical extraction.

**Figure 5:**
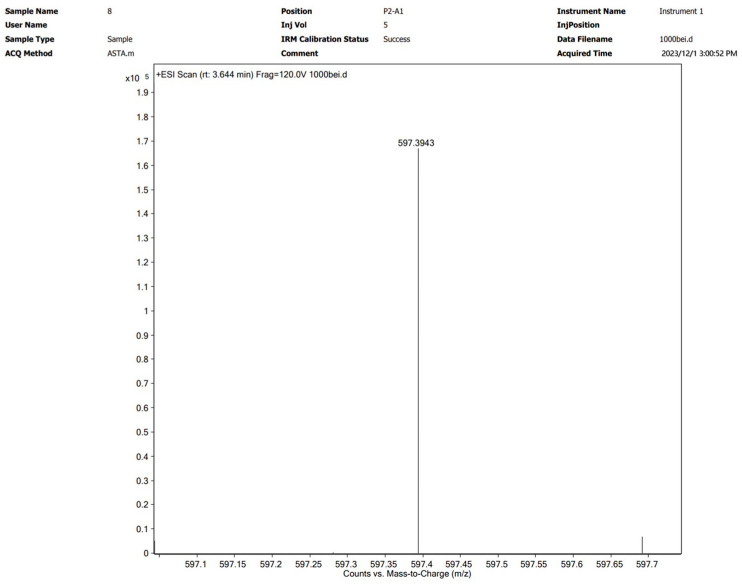
Mass spectrometry spectrum of an astaxanthin sample from *P. rhodozyma* after subcritical extraction.

Astaxanthin was extracted via the optimal method described in Section 3.2 and subsequently characterized by UPLC-Q-TOF MS. The analytical results are shown graphically in Figure 4 and Figure 5. The [M+H]^+^ molecular ion peak at *m*/*z* 597.3943 corresponds to astaxanthin, which was confirmed to be the major bioactive constituent in the subcritical extraction sample.

### 2.5. Detection of Astaxanthin Samples by Liquid Chromatography

**Figure 6:**
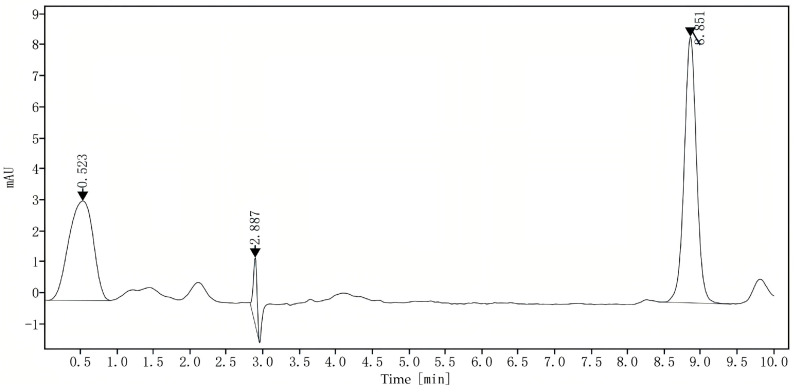
HPLC chromatogram of an astaxanthin sample from *P. rhodozyma* after subcritical extraction.

Following the protocol outlined in Section 3.5.4, the content of astaxanthin (extracted according to the optimal method described in Section 3.2) derived from the subcritical extraction of *P. rhodozyma* was quantified. The resulting chromatogram shown in Figure 6 provides a visual representation of the profile of astaxanthin from the tested samples. The filtrate was analyzed by liquid chromatography, yielding a peak area (X) of 100.787. The concentration of astaxanthin was precisely calculated to be 540.00 mg/L via the standard curve equation.

## 3. Materials and Methods

### 3.1. Main Instruments and Reagents

A 10 L glass bioreactor (BIOTECH-10JGY) was purchased from Shanghai Baoxing Biological Equipment Engineering Co., Ltd., (Shanghai, China); a 50 L bioreactor (BIO-SDQR-2000S) was purchased from Anhui Said Qirui Biotechnology Co., Ltd. (Xuancheng, China); and a scale with an accuracy of one hundred thousand was used (Mettler Toledo ME55). Additionally, high-performance liquid chromatography (HPLC) analysis was performed with an Agilent 1260 Infinity II system, and ultrahigh-performance liquid chromatography-quadrupole time-of-flight mass spectrometry (UPLC-Q-TOF MS) analysis was performed with an Agilent 6545 Q-TOF LC/MS system and an Agilent C18 column (4.6 × 250 mm, 5 µm). The subcritical fluid extraction equipment was model CBE-5L.

The following materials were procured from Aladdin Reagent Co., Ltd. (Shanghai, China): astaxanthin standard (lot# J2227049), lactic acid, dimethyl sulfoxide (DMSO), sodium hydroxide (NaOH; analytical reagent, AR), methanol (HPLC grade), and *β*-mannanase (lot# G303580, 50 U/mg). *β*-Glucanase (lot# 9025-70-1, 50 U/mg) was obtained from Rhawn Reagent Co., Ltd., (Shanghai, China).

### 3.2. Strains and Cultivation Conditions

*P. rhodozyma* CK68 was preserved in our laboratory. The yeast was maintained on yeast malt (YMA) agar slants containing 10.0 g/L glucose, 5.0 g/L tryptone, 3.0 g/L malt extract, 3.0 g/L yeast extract, and 20.0 g/L agar. The cells were cultured on the yeast malt agar (YMA) medium at 25 °C for 6 days and then stored at 4 °C.

Shake flask seed solution preparation: The *P. rhodozyma* was inoculated into the culture medium under aseptic conditions at precisely 22 °C, with agitation at 200 rpm for 24 h of incubation.

The first-grade seed mixture was prepared as follows: the seed mixture from the shake flask was inoculated into a first-grade seed tank (10 L) at a ratio of 10% and incubated for 24 h at 180 rpm and 22 °C with 1.2 m^3^/h aeration.

Fermentation culture: When sufficient yeast cells with a certain morphology were obtained in the primary seed mixture, the mixture was inoculated into a 50 L bioreactor (BIO-SDQR-2000S, Anhui Said Qirui Biotechnology Co., Ltd., Xuancheng, China) containing fermentation medium at a volume ratio of 10% for fermentation at 250 rpm and pH 6.0 with 2.4 m^3^/h aeration. After 22 h of fermentation, the dissolved oxygen content decreased and was subsequently maintained at approximately 25%. The residual glucose concentration in the fermentation broth was maintained at 2 g/L by the addition of 54% glucose concentrate. After 64 h, the tank was maintained under 50% dissolved oxygen at a pH of 5.0. After fermentation for 84 h, 10^9^ *P. rhodozyma* cells/mL were harvested.

### 3.3. P. rhodozyma Cell Wall Disruption

#### 3.3.1. Lactic Acid Method

One milliliter of *P. rhodozyma* fermentation broth was transferred into a 10 mL centrifuge tube and centrifuged at 8000 rpm for 2 min. Afterward, the supernatant was discarded, 0.6 mL of 6 mol/L lactic acid was added, and the mixture was incubated in a 30 °C thermostatic water bath for 5 min to disrupt the cell wall [20]. 

#### 3.3.2. DMSO Method

Aliquots of 1 mL of the *P. rhodozyma* fermentation broth were transferred to 10 mL centrifuge tubes and centrifuged at 8000 rpm for 2 min. The supernatant was then removed, and 3 mL of DMSO, preheated to 60 °C, was added to the pellet. The suspension was mixed with a mechanical pipette and incubated in a water bath at 50 °C for 5 min [21].

#### 3.3.3. NaOH Method

The *P. rhodozyma* fermentation broth (10 mL) was removed by aspiration, placed in a 50 mL centrifuge tube, and centrifuged at 8000 rpm for 5 min. The supernatant was discarded, and the pellet was resuspended in 10 mL of deionized water. The mixture was homogenized with a mechanical pipette. Then, 30% (by mass) of NaOH solution was added to adjust the pH to 11.8. The suspension was subsequently subjected to a cell wall disruption treatment at 30 °C for 3 h or 40 °C for 2 h in a thermostatically controlled water bath [22].

#### 3.3.4. Enzymatic Methods

*β*-Mannanase method: A 10 mL aliquot of *P. rhodozyma* fermentation broth was transferred to a 50 mL centrifuge tube, followed by the addition of 1 mg of *β*-mannanase powder (50 U/mg). The mixture was incubated in a 38 °C water bath for 72 h.

*β*-Glucanase method: A 10 mL sample of *P. rhodozyma* fermentation broth was placed in a 50 mL centrifuge tube, and 1 mg of *β*-glucanase (50 U/mg) was added. Enzymatic hydrolysis was conducted for 72 h in a 38 °C water bath.

Composite enzyme method: A 10 mL sample of *P. rhodozyma* fermentation broth was placed in a 50 mL centrifuge tube, and 0.5 mg of *β*-glucanase (50 U/mg) 0.5 mg of *β*-mannanase powder (50 U/mg) was added. Enzymatic hydrolysis was conducted for 72 h in a 38 °C water bath [23].

### 3.4. Subcritical Fluid Extraction

N-Butane was selected as the solvent for the extraction process. After analyzing the results (which were unpublished data from our laboratory) from preliminary single-factor experiments, response surface methodology (RSM) was employed to optimize the extraction conditions following the Box–Behnken design. This approach adopted a three-factor, three-level experimental layout, where the independent variables A, B, and C represented the extraction temperature, frequency of extraction, and duration of extraction, respectively. The dependent variable, Y, corresponded to the astaxanthin content and was quantified by HPLC. The codes for the factors and levels are listed in Table 5. The experimental data were subjected to statistical analysis and optimization by Design Expert software (version 8.0).

### 3.5. Detection of Astaxanthin Content

#### 3.5.1. Preparation of the Astaxanthin Standard Mother Liquor

Precisely 5.00 mg of astaxanthin standard was accurately weighed and dissolved in dimethylformamide. The solution was transferred to a 100 mL volumetric flask to generate a 50 mg/L solution after dilution. The solution was subsequently stored at 4 °C for subsequent use. The astaxanthin mother liquor was initially diluted with chromatographic-grade methanol. A series of astaxanthin standard solutions with concentrations of 0, 0.25, 0.5, 1, 2, 4, 8, and 16 mg/L were subsequently prepared.

#### 3.5.2. Liquid Chromatography Conditions

The method was based on previous research [24,25] and has been improved by delineating specific parameters for the liquid chromatography conditions, as detailed henceforth. The mobile phase consisted of 95% methanol and 5% ultrapure water and was operated at a flow rate of 1 mL/min. The chromatographic column used was an Agilent EC-C18 column (4 µm, 4.6 × 150 mm), which was maintained at 30 °C. Finally, the injection volume was 10 µL, and the DWD detection wavelength was 478 nm. 

#### 3.5.3. Construction of the Astaxanthin Standard Curve

The prepared astaxanthin standard solutions were filtered through a 0.22 µm organic membrane to ensure sterility and particle removal. The filtrate was subsequently subjected to HPLC analysis under the chromatographic conditions outlined in Section 3.5.2. This analysis yielded the corresponding peak area X, which can be used to determine the astaxanthin concentration.

The peak area data for astaxanthin, which corresponded to a particular standard concentration, were subjected to linear regression analysis to construct a standard curve and establish a quantitative relationship between the peak area and astaxanthin concentration, as follows:

Y = kX + b (R^2^ ≥ 0.99)

where Y represents the standard concentration (mg/L), X represents the peak area of astaxanthin, k represents the slope of the standard curve, and b represents the intercept.

#### 3.5.4. Astaxanthin Content Determination

A 5 µL aliquot of the products obtained from the *P. rhodozyma* liquid by subcritical extraction was diluted 1000-fold with chromatographic-grade methanol. The diluted sample was subsequently filtered through a 0.22 µm organic filter membrane to ensure sample clarity and eliminate any particulate matter. The resultant peak areas, designated X, were determined by HPLC using the chromatographic conditions outlined in Section 3.5.2. These detected peak areas were subsequently employed to calculate the astaxanthin concentration for each sample analyzed (Y).

#### 3.5.5. UPLC-Q-TOF MS Analysis

The products obtained by subcritical extraction were further characterized by UPLC-Q-TOF MS. The mass spectrometry parameters were as follows: a Dual AJS ESI ion source, a capillary current of 0.088 µA, a desolvation temperature of 350 °C, a source voltage of 4 kV, and a mass scan range of 20–1100 *m*/*z*. Chromatographic separation was performed isocratically, with a mobile phase consisting of 5% phase A (ultrapure water) and 95% phase B (0.1% formic acid). The resulting mass spectra, which corresponded to distinct temporal peaks, were acquired and analyzed for the presence of the characteristic [M+H]^+^ ion of astaxanthin at *m*/*z* 597.3938 to confirm the identity of the separated compound [26,27,28,29].

## 4. Discussion

The cellular structure of *P. rhodozyma* consists of cell walls, cell membranes, nuclei, mitochondria, the endoplasmic reticulum, the cytoplasmic matrix, and vacuoles. The substantial thickness of the cell wall severely hinders the availability of astaxanthin, although it can also protect astaxanthin from degradation [30]. Therefore, breaking the cell walls of *P. rhodozyma* is critical for enhancing the bioavailability of astaxanthin.

A variety of techniques have been implemented to disrupt yeast cell walls, including acidic [31] and lactic acid [20], DMSO [21,32,33], NaOH [22], enzymatic [23,34,35], and subcritical extraction approaches [36,37]. The DMSO method has been noted for its efficacy when applied at 45 °C for 40 min, yielding an astaxanthin output of 4.42 mg/g [21]. Choi et al. [38] and Huang et al. [32] delineated the optimal conditions for DMSO application, which corroborated our findings and resulted in effective cell wall breakdown. Despite the promising results yielded by DMSO and other organic solvents, concerns regarding their toxicity and environmental safety cannot be overlooked. Conversely, the NaOH method has been reported to achieve 80% cell wall disruption with minimal loss of pigment [39]. However, the implementation of the NaOH method is limited by operational constraints and potential environmental consequences stemming from effluent discharge.

Enzymatic methods, which employ *β*-glucosidase and *β*-mannanase, have been utilized to target the polysaccharide constituents of the yeast cell wall, achieving 60% wall disruption efficiency, which represents a significant advancement over prior methodologies [40,41]. Harith et al. [23] employed a protease and mannanase composite enzyme, which resulted in nearly 90% cell wall disruption. However, in this study, the amount of astaxanthin produced by yeast via enzymatic hydrolysis to disrupt the cell wall was lower than that produced via lactic acid fermentation.

Lactic acid treatment enhances cell permeability, thereby facilitating the extrusion of intracellular components and increasing extraction efficiency. The optimal parameters for this method have been identified as 6 mol/L lactic acid, a temperature of 30 °C, a 5 min treatment duration, and an acid-to-substrate ratio of 15 mL/g, which collectively produce pronounced cell wall disruption. Therefore, in this study, the lactic acid method was selected on the basis of its superiority in terms of cell wall disruption and astaxanthin yield, which are the most promising outcomes.

In this study, an Agilent column was used at a temperature of 30 °C, which aligns with established parameters for astaxanthin and carotenoid detection [42]. Additionally, isocratic separation was used in this study to make the operation more convenient [43,44]. The main substance obtained from subcritical extraction was identified as astaxanthin by combining liquid chromatography and mass spectrometry and astaxanthin quality counts [45,46,47].

In this experiment, a response surface optimization design was used to determine the extraction temperature, extraction frequency, and extraction time as the influencing factors. Within a certain temperature range, higher extraction temperatures correlated with improved extraction efficiency. Nevertheless, exceeding this optimal temperature range could result in astaxanthin oxidation. The yield of astaxanthin is positively correlated with the frequency of extraction; however, an increase in the number of extraction cycles results in a progressive decline in the efficiency of the process, thereby reducing its economic viability in terms of time. Furthermore, although increasing the duration of extraction can increase the quantity of astaxanthin extracted, the law of diminishing returns is applicable, with subsequent extractions yielding progressively less astaxanthin. Such an extended extraction duration also incurs additional costs, necessitating careful consideration of the balance between extraction time and economic feasibility when optimizing the extraction protocol [48,49]. Therefore, through software analysis combined with experimental data, the optimal extraction conditions were determined as an extraction temperature of 41 °C, number of extractions 2, and an extraction time of 46 min.

Using supercritical fluid extraction, Woźniak et al. extracted four furanocoumarin-rich plant matrices [50], Tyśkiewicz successfully extracted phenolic compounds from lavender flowers [51], and Miękus et al. obtained carotenoids from carrots [52]. Supercritical CO_2_ extraction has the advantages of fast extraction duration, high efficiency, easy control of the operating parameters, and low energy consumption [53,54]. However, the supercritical CO_2_ method requires high pressure and comes with a certain degree of danger and high costs and equipment requirements, and its use in large-scale production is difficult [55,56,57,58,59,60]. In contrast, subcritical extraction technology can be utilized at room temperature and pressure; thus, it can be widely used to extract natural products [61]. In our study, a relatively high concentration of astaxanthin was also obtained from *P. rhodozyma* by subcritical extraction after the process was optimized by software simulations.

## 5. Conclusions

The HPLC analysis suggested that the lactic acid method could most effectively disrupt the cell walls of *P. rhodozyma*. The use of a subcritical extraction method with specific parameters (extraction temperature of 41 °C, extraction frequency of 2, and extraction time of 46 min) facilitated the effective extraction, concentration, and enrichment of astaxanthin from *P. rhodozyma*. The results here provide a basis for obtaining astaxanthin for high-value applications.

## Figures and Tables

**Table 1:** Amounts of astaxanthin obtained by different *P. rhodozyma* wall disruption methods.

Sample	Astaxanthin Content (mg/L)
Lactic acid method	474.32
DMSO method	102.65
NaOH method (30 °C, 3 h)	97.35
NaOH method (40 °C, 2 h)	105.34
Enzymatic method: *β*-mannanase	82.08
Enzymatic method: *β*-glucosidase	78.80
Enzymatic method: composite enzyme	78.09

**Table 2:** Box–Behnken experimental design and results.

Serial Number	A	B	C	Y
	Extraction Temperature (°C)	Extraction Frequency (Times)	Extraction Time (min)	Astaxanthin Content (mg/L)
1	30.00	2.00	30.00	111.21
2	50.00	2.00	30.00	202.34
3	30.00	3.00	45.00	223.62
4	40.00	3.00	30.00	184.42
5	40.00	2.00	45.00	426.78
6	40.00	2.00	45.00	380.23
7	40.00	1.00	60.00	234.84
8	40.00	2.00	45.00	409.37
9	40.00	2.00	45.00	398.44
10	30.00	1.00	45.00	123.04
11	50.00	3.00	45.00	231.19
12	40.00	1.00	30.00	188.57
13	40.00	3.00	60.00	207.65
14	50.00	1.00	45.00	221.99
15	40.00	2.00	45.00	440.65
16	50.00	2.00	60.00	205.56

**Table 3:** Box–Behnken experimental design and results.

Serial Number	A	B	C	Y
	Extraction Temperature (°C)	Extraction Frequency (Times)	Extraction Time (min)	Astaxanthin Content (mg/L)
1	30.00	2.00	30.00	111.21
2	50.00	2.00	30.00	202.34
3	30.00	3.00	45.00	223.62
4	40.00	3.00	30.00	184.42
5	40.00	2.00	45.00	426.78
6	40.00	2.00	45.00	380.23
7	40.00	1.00	60.00	234.84
8	40.00	2.00	45.00	409.37
9	40.00	2.00	45.00	398.44
10	30.00	1.00	45.00	123.04
11	50.00	3.00	45.00	231.19
12	40.00	1.00	30.00	188.57
13	40.00	3.00	60.00	207.65
14	50.00	1.00	45.00	221.99
15	40.00	2.00	45.00	440.65
16	50.00	2.00	60.00	205.56

**Table 4:** Regression equation coefficients and significance test.

Source	Sum of Squares	df	Mean Square	F Value	*p*-Value	
Model	182,945.80	9.00	20,327.31	30.26	<0.0001	significant
A-extraction temperature	6272.00	1.00	6272.00	9.34	0.02	
B-extraction frequency	780.13	1.00	780.13	1.16	0.32	
C-extraction time	2415.13	1.00	2415.13	3.60	0.10	
AB	2025.00	1.00	2025.00	3.01	0.13	
AC	1024.00	1.00	1024.00	1.52	0.26	
BC	132.25	1.00	132.25	0.20	0.67	
A^2^	60,808.55	1.00	60,808.55	90.52	<0.0001	
B^2^	34,809.92	1.00	34,809.92	51.82	0.00	
C^2^	57,072.76	1.00	57,072.76	84.96	<0.0001	
Residual	4702.45	7.00	671.78			
Lack-of-fit	2503.25	3.00	834.42	1.52	0.34	not significant
Pure error	2199.20	4.00	549.80			
Cor total	187,648.20	16.00				

**Table 5:** Experimental factors and levels for the response surfaces.

Level	Factor		
	A	B	C
	Extraction Temperature (°C)	Extraction Frequency (Times)	Extraction Time (min)
−1	30	1	30
0	40	2	45
1	50	3	60

## Data Availability

The data are available in public sources.
